# Address of the Chairman of the Section on Stomatology of the American Medical Association, St. Paul, Minn., June, 1901

**Published:** 1901-07

**Authors:** R. R. Andrews


### ADDRESS OF THE CHAIRMAN OF THE SECTION ON STOMATOLOGY OF THE AMERICAN MEDICAL ASSOCIATION, ST. PAUL, MINN., JUNE, 1901.[1]

BY R. R. ANDREWS, A.M., D.D.S.

In reviewing some of the more important matters which have been presented to our profession during the past year, it seems to me that the symposium on Dental Education presented before this Section at its meeting is one of considerable importance as an educational factor. The time was ripe for such a discussion, and we are already beginning to see its good results. At the beginning of this twentieth century we find that advanced conditions call for a higher educational and professional standard. Some of our schools are already recognizing this, and have decided that another year should be added to their course of study, making it one of four years, and they also have under consideration the requirement of a degree in letters, or its equivalent, for entrance to their schools. Necessity for such action arises from the fact that this is an age of progression; the material development and enterprise of the time is irresistible. The man who would succeed in a profession must now grasp every legitimate opportunity. His equipment should be larger and his education broader than formerly. Success in the future is to come to him who shall be educated to do a thing as well as it is possible to do it; that is, he must become a specialist in some department of his chosen profession. I have been informed that President Eliot, of Harvard University, after reading the symposium on Dental Education, writes to our secretary that he is favorable to the establishment of a Medical University, where all the medical specialties, including dentistry, shall be taught under one head and a common degree be given to all. President Capen, of Tufts College, writes, me, under date of February 21, 1901: "I have received your pamphlet, containing an account of the proceedings of the Section on Stomatology of the American Medical Association. I have not only read your own paper with great interest and profit, but have examined the other papers sufficiently to get the drift of the sentiment of the Association. I was sur-

[1] Abstract prepared from official report.

prised to find it so strongly in accord with my own views. I may add that I find now, both in our medical and dental faculties, an opinion strongly favoring a four years' course for dental students as well as medical students, and the common degree of M.D. for all.

Some of the criticisms against this common degree come from eminent professors in our special schools, who are earnest, able, and conscientious men, sincerely believing that if we are to educate our students in medicine, they will become theorists, as are most of the educated dentists from the schools of Europe, and that the practical technical training, which in the past has made the name of the American dentist honored all over the world, will no longer be acquired; that under this new system American dentists will become thinkers rather than operators, scientists rather than practical men.

Such opinions are to be respected, but, on the other hand, the four years' course could be systematically arranged to give technical training fully equal to that given to-day in our best special schools, together with the necessary medical education that shall warrant the common degree. We should demand the same medical education that is required in other medical specialties, with all, or more, of the practical training that shall teach the hand as well as the head.

Another subject nearly as important is in relation to the many problems now before our profession, and as to the best methods of solving them. This has recently been considered by the Dental Society of the State of New York, and should receive our cordial co-operation. In considering these problems, will it be wisest to have the individual investigator proceed in his own way, paying his own expenses in solving our problems, or will it be better for national or State societies to establish commissions, whose duty shall be to oversee and encourage research work, propose problems to be worked out, and also have it in their power to furnish the necessary funds for carrying out this work? If such a commission were established, is it not reasonable to suppose that the profession would be greatly benefited and result in a more uniform practice? Such a commission should be composed not merely of eminent practitioners, but of men of judicial minds and training, whose duty should be to collect the facts, weigh the evidence, and give rulings on disputed points.

Among the students in our special schools there are always to be found a few who have the inborn faculty of investigation.

While building up a practice they give a very considerable amount of time in solving some of the scientific problems which appeal to them. Some of these men become well known to the profession, others do not. There is a very considerable expense attending many of these investigations, and research work would be better done if they could afford suitable apparatus. Work of this nature is of value to the whole profession, and it seems as though it would be wise to establish the commission suggested. Investigators could then devote a certain portion of time to solving problems and receiving therefore adequate compensation. Our schools also should encourage research work, and a department for it should be established where men who are capable should be given the opportunity to work along lines congenial to them. This may lead to a career of much value to themselves and of great good to their profession.

There are one or two important matters which I think it would be wise to consider in regard to the subject of State Boards of Dental Examiners, which is to be presented in a series of papers for our consideration and discussion at this session. This subject is an important one and will, I am sure, receive careful attention in broad and generous criticism. We are all mindful of the fact that in many of the States the board of dental examiners has been largely instrumental in raising the professional standard and in influencing in no small degree the necessity for a higher educational qualification in our special schools. With all this, there is yet something to be desired. An appointment to the board should never be a political gift. The nomination of suitable persons to fill vacancies on such a board should be made to the appointing power by nominating committees from the State societies, elected for this purpose. This plan works admirably in Massachusetts. A standard should be established by the National Board of Dental Registration for the boards of the different States, that shall make it possible for a person who has once passed a successful examination to practise his profession in any other State without further examination. And again, in justice to the older members of our profession, discretionary powers should be given the boards of the several States to guide them in granting certificates to those who have for years been active in honorable practice and who are not, by reason of advancing years, fitted to pass the same examination as should be demanded of recent graduates.

In the department of Prophylaxis the future is bright with possibilities. Recent investigation is opening up a field that gives promise soon of successful treatment. J. Leon Williams, in his brilliant paper on "The Bacteria of the Human Mouth," has demonstrated beyond a doubt that there exists upon the teeth of persons predisposed to caries a hardened gelatinous film, which is a culture medium for the growth of myriads of micro-organisms. This film he calls the "gelatinous microbic plaque." The organisms found here are largely the leptothrix racemosis, together with the leptothrix buccalis. The leptothrix racemosis is, like the leptothrix buccalis, a threadlike form, but differs in having myriads of spores growing from an extremity, where they multiply with marvellous rapidity. They are benign or malignant according to their environment, and the saliva in an abnormal condition is the exciting cause of dental caries.

Professor J. P. Michaels, of Paris, who has given the results of his recent brilliant investigations of human saliva at the International Dental Congress, tells us that, as a result of overactivity of the liver, a superabundance of glycogen may be found in the saliva, giving it an alkaline reaction; but coming in contact with the ptyalin which is always present in the saliva, a reduction occurs of the glycogen, first to glucose and then to lactic acid, which unites with the lime of the tooth. He has experimented with ptyalin and glycogen (either alone giving a neutral solution), and put them in separate bottles containing distilled water, then connected the liquids in the two bottles with a piece of cord, which formed a loop around and in contact with a chalk crayon. By capillarity the liquids came together at the chalk, which was dissolved away wherever touched by the cord. He asks, "Does not this tend to show how decay of the teeth may occur in an alkaline saliva and independent of microbic influences?"

Michaels has also shown that exciting and pathological conditions can be found and are positively recognizable in the saliva. He has been able to determine the histochemical condition of saliva of those who are not affected with carious teeth, and suggests that nutrition may establish vital conditions so desirable in securing immunity from decay.

A paper of special interest on treatment to prevent the decay of the teeth has been presented by Dr. D. D. Smith, of Philadelphia, and should command our serious attention when considered from the stand-point of recent investigation. The simple method is to place in a suitable carrier a piece of orange-wood sharpened to a chisel-point for using on the flat surfaces of the teeth, and another sharpened to a pencil-point to be used between them. These points are to be renewed as often as they become frayed. Before using they are dipped in water, and then pumice, and with them the surfaces of the teeth are polished up into the festooning of the gum, using enough force to stimulate, and cleansing what may appear to be clean teeth, going over all surfaces thoroughly, outside, inside, between, and on the occlusal surfaces. This work should be done as thoroughly as it is possible to do it, leaving the teeth in a polished and smooth condition. Such action has a stimulating influence, and under this stimulation the internal vital function seems aroused to new activities. Treatment is to be given once a month; the patients are instructed to thoroughly care for their teeth daily, using fine salt once a week. It produces stimulation of the gum-tissue, of the alveolar tissue, and of the whole oral structure. This simple but vigorous treatment, by removing the gelatinous microbic plaque which Williams describes, induces the most attractive and apparently most perfect condition of the mouth attainable.

Another subject of particular interest to this body is the law creating a dental service in the United States army, enacted by Congress since our last Session. Although the law as enacted is not all that we asked for, it is an entering wedge which may result in more satisfactory legislation in the future. Dr. Maynard, of Washington, somewhere about the year 1850, was very considerably interested in the enactment of such a law, and used his influence with the President and Secretary of War to have it passed, but without success. In July, 1858, the late Dr. H. J. McKellops, of St. Louis, offered a resolution before the Western Dental Society at Quincy, Ill., with the idea of establishing such a law. It may thus be seen that the movement is not a new one, but that, with the growth of public appreciation of dental services, which is justly regarded as one of the common necessities of life, the demand has been created and recognized. Dr. John S. Marshall, of Chicago, one of our most esteemed and valued members, has been appointed President of the Examining Board of Dental Surgeons of the United States army. And he is a man in every way eminently fitted for this position. The high standard which is demanded for the appointment of contract dental surgeons under the new law may be recognized from the fact that out of fourteen candidates, only two successfully passed the examinations. Dr. Marshall, in a communication, says, "It is to be hoped, therefore, that our dental schools will not recommend any young men to come before this board who are not thoroughly well qualified, theoretically and practically, in all the branches comprising the curriculum of our best dental schools."

In the department of Biology, science has made some of her most remarkable advances. The work of the investigator has opened up new vistas of relations, of which many of us have never dreamed. In an article entitled "Aspects of Recent Biological Research," recently published in the International Dental Journal, from which time will allow me only the very briefest quotations, Professor E. B. Wilson, of Columbia University, says,—

"Chemical analysis proves that protoplasm is not a single chemical substance, but a highly complex mixture of many highly complex organic compounds, which undergo continual chemical transformations,—indeed, a living cell is doubtless the area of the most complex chemical operations taking place in nature. . . . In the fertilization of the egg, a single cell derived from the father (the spermatozoon) unites with a single cell (the egg) derived from the mother, while the paternal nucleus unites with the maternal. By continued division of the single cell thus formed, arise all the cells of the body. If, now, we examine the details of this process, we find one remarkable difference between the egg and spermatozoon,—namely, that while the two contribute equally to the nucleus of the fertilized germ, the whole, or very nearly the whole, of the remaining cell-substance (protoplasm) is supplied by the egg. We have thus a substantial basis for the conclusion that the protoplasm of the embryo is derived wholly or mainly from the mother, while the nuclei are equally derived from both parents. If this result be placed beside those derived from microscopical vivisection, we gain for the first time a clear, if superficial, view of the mechanism of inheritance, and can form a definite mental picture of the manner in which it is effected. . . . As the paternal and maternal nuclei approach each other within the egg, each undergoes a complicated metamorphosis and finally resolves itself into a number of rod-like or worm-like bodies known as chromosomes, which are exactly equal in number and similar in form in the two. At this period, therefore, the egg no longer contains two nuclei, but in their place two precisely similar groups of chromosomes, which are respectively paternal and maternal in origin. As the egg undergoes its first division to form the first two cells of the embryo, every chromosome in each group splits lengthwise into two exactly similar halves, which separate and pass one to each of the daughter cells. Each of the latter, therefore, receives two similar groups of daughter chromosomes, paternal and maternal, which are exactly reduplicated in the other cell, and from these two groups in each cell is built up a daughter nucleus, shown by its mode of origin to be equally derived from both parents. At the second division the chromosomes reappear again split lengthwise, and the halves are again equally distributed to the daughter nuclei, and so on throughout the entire life of the animal."

Among the other facts noted in this article by Professor Wilson, is the fact that " The egg may be fertilized by chemical stimulus without participation of the male element. If, for example, the eggs of sea-urchins be allowed to develop in sea-water containing a very slight excess of potassic chloride, the development of the embryo is greatly altered, no skeleton is formed, and a larva results which, though living and vigorous, is of widely different form from the normal ones. If, in place of potassic chloride, lithium chloride be added to the water, the changes are still more remarkable, the embryo never infolding the cells which normally give rise to the alimentary canal, but developing, as it were, inside out. These monstrous forms are of course incapable of nourishing themselves, and ultimately perish, but the result is of high interest as opening the possibility of creating wholly new organic forms by varying slightly the conditions of development. . . . It is certain that new growths of the highest interest relating to the chemical conditions in living matter may be looked for along the lines of research thus opened. . . . Experiments on insects, frogs, and rotifers have already given good ground for the conclusion that sex is in these cases determined by conditions of nutrition, which again in the long run are reducible to chemical conditions. The possibility is thus opened that we may yet succeed not only in fertilizing the egg by chemical means, but also in rendering the organism, male or female, by analogous methods."

In closing I regret that I have not been able to offer any research work, and that the time allotted for this address will not permit the review of many important papers offered to the profession during the last year. I would suggest that the leading symposium for our next year's session be Prophylaxis. Our Section is well fitted to consider this subject, and the recent investigations should stimulate us to earnest and serious work.

The Section is now open for its regular proceedings.

